# Isolated HIV-1 core is active for reverse transcription

**DOI:** 10.1186/1742-4690-4-77

**Published:** 2007-10-24

**Authors:** David Warrilow, Deborah Stenzel, David Harrich

**Affiliations:** 1 Division of Immunology and Infectious Disease, Queensland Institute of Medical Research, Brisbane, Queensland, 4006, Australia; 2 Analytical Electron Microscopy Facility, Queensland University of Technology, Gardens Point Campus, Brisbane, Queensland, 4001, Australia

## Abstract

Whether purified HIV-1 virion cores are capable of reverse transcription or require uncoating to be activated is currently controversial. To address this question we purified cores from a virus culture and tested for the ability to generate authentic reverse transcription products. A dense fraction (approximately 1.28 g/ml) prepared without detergent, possibly derived from disrupted virions, was found to naturally occur as a minor sub-fraction in our preparations. Core-like particles were identified in this active fraction by electron microscopy. We are the first to report the detection of authentic strong-stop, first-strand transfer and full-length minus strand products in this core fraction without requirement for an uncoating activity.

## Findings

Deoxyribonucleotides added directly to HIV-1 virions are incorporated into reverse transcription products [1-4]. This process, which is reported to disrupt the structure of the core in virions [5], is referred to as natural endogenous reverse transcription (NERT). Restructuring of the core also occurs post-infection when the core enters the cytoplasm after fusion of the viral envelope and is referred to as uncoating [6]. One commonly accepted interpretation of NERT is that the observed virion disruption is analogous to uncoating, and uncoating may be a requirement for formation of an active reverse transcription complex (RTC) (reviewed in [7]).

An alternative corollary of the ability of intact virions to generate reverse transcription products is that cores purified from virions should be capable of reverse transcription. Whilst purified cores have been shown to contain reverse transcriptase [8-15], there is just one report of cores generating authentic reverse transcription products, but only when complemented with an "uncoating activity" from activated lymphocytes [16]. The question of the biochemical state of virion core is of particular interest in the light of recent reports of reverse transcription in cores *in vivo *[17], and is important for our understanding of early replication events. To explore this controversial question, we used a modification of a commonly used method of core purification. We demonstrated that cores were able to generate authentic RT products without a requirement for an uncoating activity, as described below.

### Core fractions have reverse transcription activity

Isolation of morphologically intact cores from HIV-1 particles has been reportedly improved by "spin-thru" methods [8,18]. The principle of the method is that virions are delipidated by brief sedimentation through a detergent layer (0.03% Triton X-100). Free cores are separated from virions and debris by subjecting them to equilibrium gradient sedimentation on a continuous 20–60% Optiprep density gradient for 20 h; cores sediment to the dense layers (1.24 – 1.28 g/ml).

**Figure 1:**
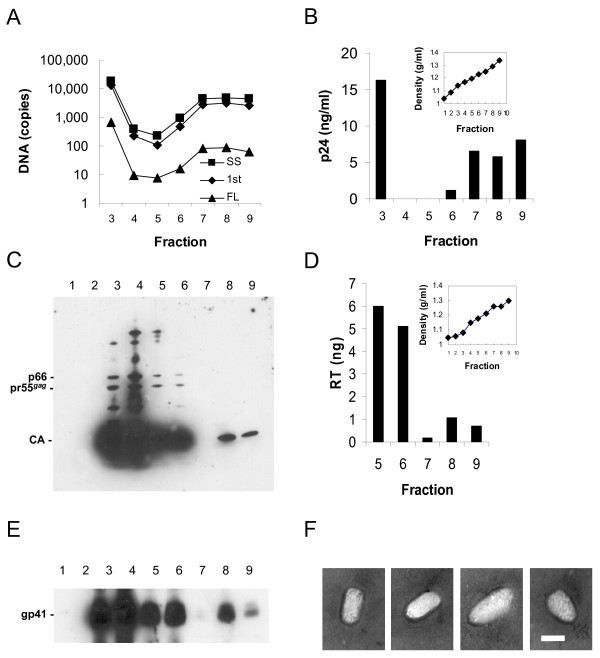
**Analysis of core fractions**. (A) Endogenous reverse transcriptase activity: strong-stop (squares), first-strand transfer (diamonds) and full-length targets (triangles) are shown. (B) p24 ELISA on fractions; inset shows the density of fractions calculated from weight (fractions 3–9 only are shown). Viral proteins were detected in HIV-1_NL4.3 _equilibrium gradient fractions 1–9 by western analysis using (C) anti-HIV-1 polyclonal antibody, (D) colorimetric reverse transcriptase ELISA using homoploymeric template (fractions 5–9 only are shown), and (E) anti-gp41 antibody. (F) Negative staining transmission electron microscopy of dense fractions showing four representative core-like structures. 100,000× magnification; bar indicates 50 nm. Please note, the fractions shown in A and B are from a separate preparation to those in C-E and hence fraction numbers do not directly correspond [see Additional file 1 for complete methods].

A high titre HIV_NL4.3_ virus stock was grown on CD4/CXCR4-expressing HeLa cells (MAGI) and was subsequently concentrated by centrifugation on a 20% sucrose cushion. We subjected two virus samples to 20–60% Optiprep density gradient centrifugation for 20 h: one with a detergent layer and a control without a detergent layer. Fractions were obtained and assayed for capsid by p24 ELISA and the ability to generate authentic reverse transcription products (endogenous reverse transcription or ERT activity). Interestingly, with repeated attempts we were not able to detect ERT products using core fractions prepared by brief passage through the detergent layer (data not shown; Warrilow et al., manuscript under review). However, a control preparation without a detergent layer had capsid and ERT activity in fractions 7–9 (Fig 1A, B) corresponding to the reported buoyant density of core (peak fraction 1.29 g/ml). A clear peak in activity was seen, for example, fraction 8 contained 30-fold more ERT activity than fraction 5. These three peak fractions represented 6% of total ERT activity of the fractions. This core fraction was capable of first-strand transfer, and full-length minus strand synthesis was also detectable above background (Fig. 1B). However, the signal was not sufficient to determine whether products indicating second-strand transfer had been generated (data not shown). This result was repeated in three separate experiments. Hence, a naturally occurring core fraction was capable of advanced reverse transcription.

### Western analysis and electron microscopy of core fractions

Western analysis was performed on gradient fractions to determine their composition. To provide sufficient material for analyses, a fresh equilibrium gradient scaled up approximately 20-fold was performed (Fig. 1C–F), and fractions were then analyzed by western analysis using purified anti-HIV-1 IgG (NIH AIDS Research and Reference Reagent Program). Multiple protein bands in the peak virus fractions 3 and 4 (1.08 and 1.15 g/ml, respectively) reacted with Gag proteins including capsid (Fig. 1C) as expected for intact virions. Only capsid protein was detected in the denser fractions 8 and 9 (density 1.26 and 1.30 g/ml, respectively), confirming our ELISA results. Reverse transcriptase was detected in these fractions by colorimetric ELISA using homopolymeric template (Fig. 1D); matrix was detected by western analysis using a specific monoclonal antibody (data not shown) as has been reported in other core preparations [11,13,14]; and gp41 was also detected in fractions 8 and 9 (Fig. 1E). A small amount of gp41 has been reported in cores purified using detergent [11]. In that study, gp41 was attributed to microvesicles that co-purified with the cores. This seems unlikely as microvesicles are generally less dense than core [19]. Alternatively, due to our novel virus culture method, our preparation may have contained a proportion of immature virions which are known to a have a stable association between gp41 and immature cores [20].

Transmission electron microscopy (TEM) was used to further characterize the denser fractions. Confirmation that the denser fractions of the untreated sample contained cores was obtained when numerous 80 – 100 nm cone and rod-shaped structures were observed in these fractions (Fig. 1F). No whole virions were observed. The above data are consistent with dense fractions with capsid and ERT activity which most likely contain biochemically active cores.

We are the first to report the detection of authentic strong-stop, first-strand transfer and full-length minus strand products in a core fraction. This confirms our expectations, from observations of the NERT reaction, that core is capable of reverse transcription, at least to full length minus-strand synthesis. It confirms that the enzymatic activities sufficient for reverse transcription are present in the core. Our data also support the suggestion that core may increase the effective concentration of components important for reverse transcription reaction, facilitating strand transfers and the efficiency of the overall reaction. The density of core does not sterically block polymerase elongation; however, we have no data as to the effect of elongation on core structure and it could be that the elongation of the polymerase results in shedding of capsid as suggested by the effect of NERT on virion morphology [5]. Some cellular protein, perhaps the uncoating factor, may assist the elongating complex to efficiently complete reverse transcription.

Preparation of cores without detergent treatment to remove the viral envelope would appear to be counterintuitive. Interestingly, in support of our data, capsid protein has been reported in dense fractions of virions subjected to equilibrium gradient ultracentrifugation without prior detergent treatment [21], although the reverse transcription capacity was not assessed. One explanation for the presence of cores in our samples is that virions could have been gently disrupted by our culture and purification method, as core release by damage to virions has been reported [22]. We chose to amplify virus on MAGI cells for 6 days prior to concentration on 20% sucrose cushion (see supplementary methods). This method may have been sufficiently disruptive to the envelope to result in core release.

Our data conflict with these previous observations of a requirement for an "uncoating activity" to activate reverse transcription activity [16]. It is possible that cores prepared using detergent methods require complementation by a cell factor, perhaps an uncoating activity, to be activated. In contrast, we have found cores to be active for reverse transcription, at least making DNase I-resistant full length minus-strand DNA, albeit inefficiently, without requiring capsid release.

Our isolation of active cores without detergent treatment was fortuitous and reproducible; however, the quantity of the naturally-occurring core fraction varied from preparation to preparation. We, therefore, attempted to isolate cores by a more reliable method. Due to the denaturing effects of detergent, we attempted a number of other methods (data not shown) such as freeze-thaw treatment and exposure to β-cyclodextrin, which removes cholesterol and leads to lipid bilayer breakdown [23]. To date none of these methods has resulted in reliable isolation of cores that are positive for ERT activity.

We have provided evidence for reverse transcription in a core fraction, and previous detergent experiments also suggest core structure is important for this process (Warrilow et al., manuscript under review). Whilst our data indicate a cell "uncoating activity" is not required to initiate reverse transcription or generate some late products, it is still consistent with a model in which the elongating RTC formation requires a cellular factor(s), for regulation of uncoating, or for completion of reverse transcription.

## Abbreviations

ERT, endogenous reverse transcription; MAGI, CD4/CXCR4 expressing HeLa cells; NERT, natural endogenous reverse transcription; TEM, transmission electron microscopy.

## Competing interests

The author(s) declare that they have no competing interests.

## Authors' contributions

David Warrilow conducted experiments, Deborah Stenzel assisted with the electron microscopy, and David Warrilow and David Harrich both designed experiments and wrote the manuscript. All authors have read and approved the final manuscript.

## Supplementary Material

Additional file 1 Supplementary materials and methods. Detailed materials and methods. Click here for file
