# Supplementary material for: Isolated HIV-1 core is active for reverse transcription
Source: Retrovirology. 2007 Oct 24;4:77. doi: 10.1186/1742-4690-4-77 (PMC2169257; doi:10.1186/1742-4690-4-77)
Supplement: Additional file 1 — Supplementary materials and methods. Detailed materials and methods. [file 1742-4690-4-77-S1.pdf]

Isolated HIV-1 Core is Active For Reverse Transcription

Supplementary materials and methods

Cell lines and virus culture. The MAGI CD4/CXCR4-expressing cell line (NIH AIDS Research and Reference Reagent Program) was grown in Dulbecco's modified Eagles medium (DMEM) supplemented with 10% FBS, penicillin-streptomycin, glutamine, 0.2 mg/ml geneticin, 0.1 mg/ml hygromycin B, and 1μg/ml puromycin. HEK293T cells were grown in RPMI1640 supplemented with 10% heat-inactivated fetal bovine serum (FBS) and penicillin-streptomycin. All cell lines were incubated at 37 ^{o}C in 5% CO_2. A stock of $HIV_{NL4.3}$ [1] was generated by transfection of the corresponding proviral DNA into HEK293T cells using Lipofectamine™ 2000 (Invitrogen, Carlsbad, CA, USA), according to the manufacturer's recommendations. Cell culture supernatants were removed at 48 h post-transfection, centrifuged (200 *g*, 10 min), the supernatant was filtered (0.45 μm pore size) and stored at -80 ^{o}C. To generate a MAGI cell line stock, cells were plated to give approximately 50% confluence and then incubated overnight. The cells were infected with HIV_{NL4-3} (250 ng p24) and incubated under standard conditions for 2 h. The culture medium was then removed and the cells were washed four times with sterile PBS. Fresh medium was added and the cells were incubated under standard conditions for 6 days. The supernatant was then harvested, centrifuged (1000 rpm, 10 min) and filtered (0.45 μm). Virus was concentrated by ultracentrifugation (100000*g*, 2 h, 4 ^{o}C) and resuspended in one-tenth of the original culture volume of sterile PBS, and stored at -80 ^{o}C.

The plasmids used in this study were made available through the National Institutes of Health AIDS Research and Reference Reagent Program.

Optiprep equilibrium gradient centrifugation. Optiprep (Axis-Shield) was diluted from 60% to 20% in 5% steps in buffer (5 mM Tris, pH 7.4, 20mM NaCl; 1 mM $MgCl_2$ and 0.5 mM β-ME). Optiprep was added to a centrifuge tube in layers of 400 μl, starting with the 60% layer at the bottom and progressively increasing up to 20%. The layers were allowed to diffuse at room temperature for 3.5 h to form a continuous density gradient. A layer (200 μl) containing 15% Optiprep then was placed above the gradient and a second layer (200 μl) containing 10% Optiprep was laid above this. The samples (400 μl) were placed on the top of the gradient and centrifuged (Beckman Sw60Ti, 34000 rpm, 4 ^{o}C, 20 h). Fractions (400 μl) were collected from the top of the tube. The fractions were assayed for endogenous reverse transcriptase activity, reverse transcriptase activity, p24 antigen, and density of the fraction was determined by weighing 100 μl.

ERT assays. Reverse transcription products were generated by addition of virus particles (equivalent to 10 ng p24) to a mixture (final volume of 50 μl) containing 10 mM Tris, pH 7.4, 10 mM $MgCl_2$, 0.1 mM Triton X-100, 500 U/ml DNase I and 200 μM each dNTP in RPMI1640 medium for 18-20 h. A no-nucleotide control reaction was always included. Products were extracted, once with an equal phenol:chloroform:iso-amyl alcohol (25:24:1) and once with chloroform. The extracts were ethanol precipitated, washed with 70% ethanol, dried and resuspended in 100 μl of 0.1 mM EDTA. Purified reaction products (5 μl) were added to the reaction mix containing 0.4 μM of each primer, SYBR Green I, 30U/ml Platinum Taq polymerase, 20 mM Tris-HCl pH

8.4, 50 mM KCl, 3 mM $MgCl_2$, 200 μM each dNTP, 20 U/l uracil-N-glycosylase (Invitrogen, Carlsbad, CA, USA) in a final volume of 15 μl. A no-DNA control (5 μl of 0.1 mM EDTA, pH 8.0) was also included. Standard primer sets used for amplification were: strong-stop DNA, #48(5'-d AAGCAGTGGGTTCCCTAGTTAG-3') and #73(5'-d GGTCTCTCTGGTTAGACCA-3'); first-strand transfer, #169(5'-d AGCAGCTGCTTTTTGCCTGTACT) and #187(5-d ACACAACAGACGGGCACACAC); full-length minus strand, #2(5'-d CAAGTAGTGTGTGCCCGTCTGTT) and #7(5'-d CCTGCGTCGAGAGAGCTCCTCTGG); and second-strand transfer #169(5'-d AGCAGCTGCTTTTTGCCTGTACT) and #7(5'-d CCTGCGTCGAGAGAGCTCCTCTGG). The mixes were subjected to cycling: $[50\ ^oC$, 2 min; 95 ^oC, 2 min$]_1[95\ ^oC$, 15 sec; 65 ^oC, 30 sec$]_{40}$ on a Rotor-Gene 3000™ thermocycler (Corbett) set to collect SYBR fluorescent signal after the 65 ^oC step. Copy number was determined by reference to a standard curve prepared by dilution of plasmid DNA (NL4.3 strain). A no-nucleotide control was always included and was negligible or the data were discarded.

Western analysis. For immunoblotting analyses, proteins were separated by electrophoresis on 12% SDS-PAGE. A Hybond-P (Amersham) membrane was cut to the appropriate size, hydrated in methanol, rinsed in distilled water and equilibrated in Towbin buffer (25 mM Tris, 192 mM glycine, 20% methanol). Separated proteins were transferred to membrane by semi-dry transfer using Towbin buffer at 25 V for 30 min. The membrane was blocked using 5% non-fat skim milk (NFSM) in TBS-T (150 mM NaCl; 10 mM Tris, pH 8.0; and 0.05% Tween-20) overnight at 4 ^oC. Primary antibodies used were

either human polyclonal antisera to HIV-1 (NIH AIDS Research and Reference Reagent Program) diluted 1:1500 or a mouse monoclonal antibody 5A1 to gp41 (Biodesign) diluted 1:200. Secondary HRP-conjugated antibodies were either anti-human IgG (Sigma) diluted 1:3000 or anti-mouse IgG (Zymed) diluted 1:5000. Antibodies were diluted in TBS-T with 5% NFSM and incubated with the membrane at RT for 1 h. The membrane was washed with TBS-T 3X for 10 min. Chemiluminescent substrate components (Pierce) were mixed (2 ml each) and placed on top of the membrane. The membrane with substrate was incubated for 5 min. Substrate was removed and the membrane was exposed to film.

Reverse transcriptase and p24 assays. Reverse transcriptase (Roche) and p24 colormetric assays (Zeptometrix) were commercially supplied and were used according to the manufacturer's instructions. Briefly, reverse transcriptase products were labeled with biotin and digoxigenin-modified deoxynucleotides overnight at 37 ^{o}C. Labeled products were bound to the walls of a streptavidin-coated plate and unbound material was removed by washing. Bound reaction products were detected with an anti-digoxigenin antibody conjugated to peroxidase. The plate was then washed and reaction products detected enzymatically with ABTS substrate. To detect p24, samples placed in a reaction well with bound p24 antibody in a buffer containing Triton X-100 in PBS for 2 h at 37 ^{o}C. Unbound material was removed by washing. Bound p24 was then reacted with biotin-labeled polyclonal antisera to HIV-1 for 1h at 37 ^{o}C followed by peroxidase conjugated streptavidin for a further 1 h at 37 ^{o}C, washing between each step. Antigen was detected enzymatically

with addition of tetramethylbenzidine (TMB) substrate for 30 min, after which the reaction was stopped.

Electron microscopy. Core fractions (200 µl) were fixed by addition of 0.1% glutaraldehyde in PBS (to final volume of 1 ml). The sample was incubated for 30 min at room temperature and then increased to 5 ml with PBS. Cores were concentrated by centrifugation (100000*g*, 2 h, Sw55Ti, 4 ^{o}C). The pellet was resuspended O/N in 20 µl of PBS. Samples (5 µl) were spotted onto carbon-coated 200 mesh grids, negatively stained with 1% aqueous uranyl acetate, and observed at 100,000X magnification in a JEOL 1200EX transmission electron microscope.

References

1. A Adachi, HE Gendelman, S Koenig, T Folks, R Willey, A Rabson, MA Martin: **Production of acquired immunodeficiency syndrome-associated retrovirus in human and nonhuman cells transfected with an infectious molecular clone**. *J Virol* 1986, **59**:284-91.
